# Binding properties of the anti-TB drugs bedaquiline and TBAJ-876 to a mycobacterial F-ATP synthase

**DOI:** 10.1016/j.crstbi.2022.09.001

**Published:** 2022-09-09

**Authors:** Alexander Krah, Gerhard Grüber, Peter J. Bond

**Affiliations:** a Bioinformatics Institute, Agency for Science, Technology and Research (A∗STAR), 30 Biopolis Str., #07-01 Matrix, 138671, Singapore; b School of Biological Sciences, Nanyang Technological University, 60 Nanyang Drive, 637551, Singapore; c Department of Biological Sciences, National University of Singapore, 14 Science Drive 4, 117543, Singapore

**Keywords:** F-ATP synthase, TBAJ-876, Molecular dynamics simulations, Mycobacteria, Diarylquinolines, Bedaquiline

## Abstract

Tuberculosis (TB), the deadly disease caused by *Mycobacterium tuberculosis* (*Mtb*), kills more people worldwide than any other bacterial infectious disease. There has been a recent resurgence of TB drug discovery activities, resulting in the identification of a number of novel enzyme inhibitors. Many of these inhibitors target the electron transport chain complexes and the F_1_F_O_-ATP synthase; these enzymes represent new target spaces for drug discovery, since the generation of ATP is essential for the bacterial pathogen's physiology, persistence, and pathogenicity. The anti-TB drug bedaquiline (BDQ) targets the *Mtb* F-ATP synthase and is used as salvage therapy against this disease. Medicinal chemistry efforts to improve the physio-chemical properties of BDQ resulted in the discovery of 3,5-dialkoxypyridine (DARQ) analogs to which TBAJ-876 belongs. TBAJ-876, a clinical development candidate, shows attractive *in vitro* and *in vivo* antitubercular activity. Both BDQ and TBAJ-876 inhibit the mycobacterial F_1_F_O_-ATP synthase by stopping rotation of the *c*-ring turbine within the F_O_ domain, thereby preventing proton translocation and ATP synthesis to occur. While structural data for the BDQ bound state are available, no structural information about TBAJ-876 binding have been described. In this study, we show how TBAJ-876 binds to the F_O_ domain of the *M. smegmatis* F_1_F_O_-ATP synthase. We further calculate the binding free energy of both drugs bound to their target and predict an increased affinity of TBAJ-876 for the F_O_ domain. This approach will be useful in future efforts to design new and highly potent DARQ analogs targeting F-ATP synthases of *Mtb*, nontuberculosis mycobacteria (NTM) as well as the *M. leprosis* complex.

## Highlights

• BDQ inhibits mycobacterial F-ATP synthase.
• TBAJ-876 is a BDQ analogue with improved affinity for the enzyme.
• Simulations help to structurally clarify the F_O_ domain binding sites of TBAJ-876.
• Insights will help guide development of multidrug-resistant tuberculosis drugs.

## Graphical abstract

**Figure:**
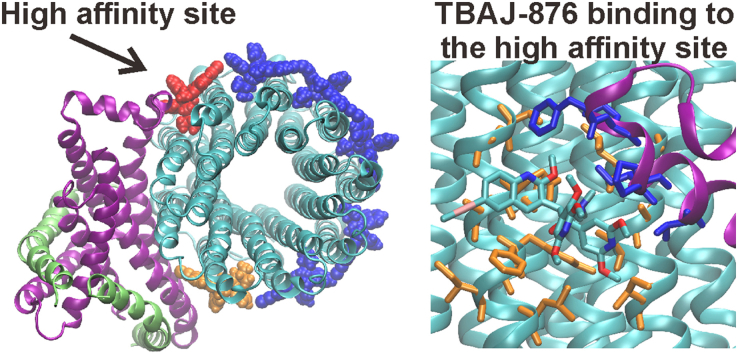

## 1 Introduction

*Mycobacterium tuberculosis* (*Mtb*) is an obligate aerobe, which is strictly oxygen dependent in order to meet its energetic demands during growth. Due to the lack of an effective fermentative process, the oxidative phosphorylation (OXPHOS) pathway is crucial to maintain redox homeostasis and the synthesis of sufficient quantities of ATP (Cook et al., 2014; Rao et al., 2008). Although mycobacteria are obligate aerobes, they can survive under low oxygen tension (hypoxia) via cell cycle exit and entry into a dormant state. Hypoxic non-replicating *Mtb* exhibits a reduced pool of ATP; this makes it finely sensitive to any further ATP depletion, and thus susceptible to drugs that target maintenance of ATP homeostasis (Rao et al., 2008). This implies that drugs which lead to inhibition of OXPHOS could shorten therapy times for drug-resistant tuberculosis, as supported by the clinical use of Sirturo® (bedaquiline, BDQ) (FDA, 2012).

**Fig. 1:**
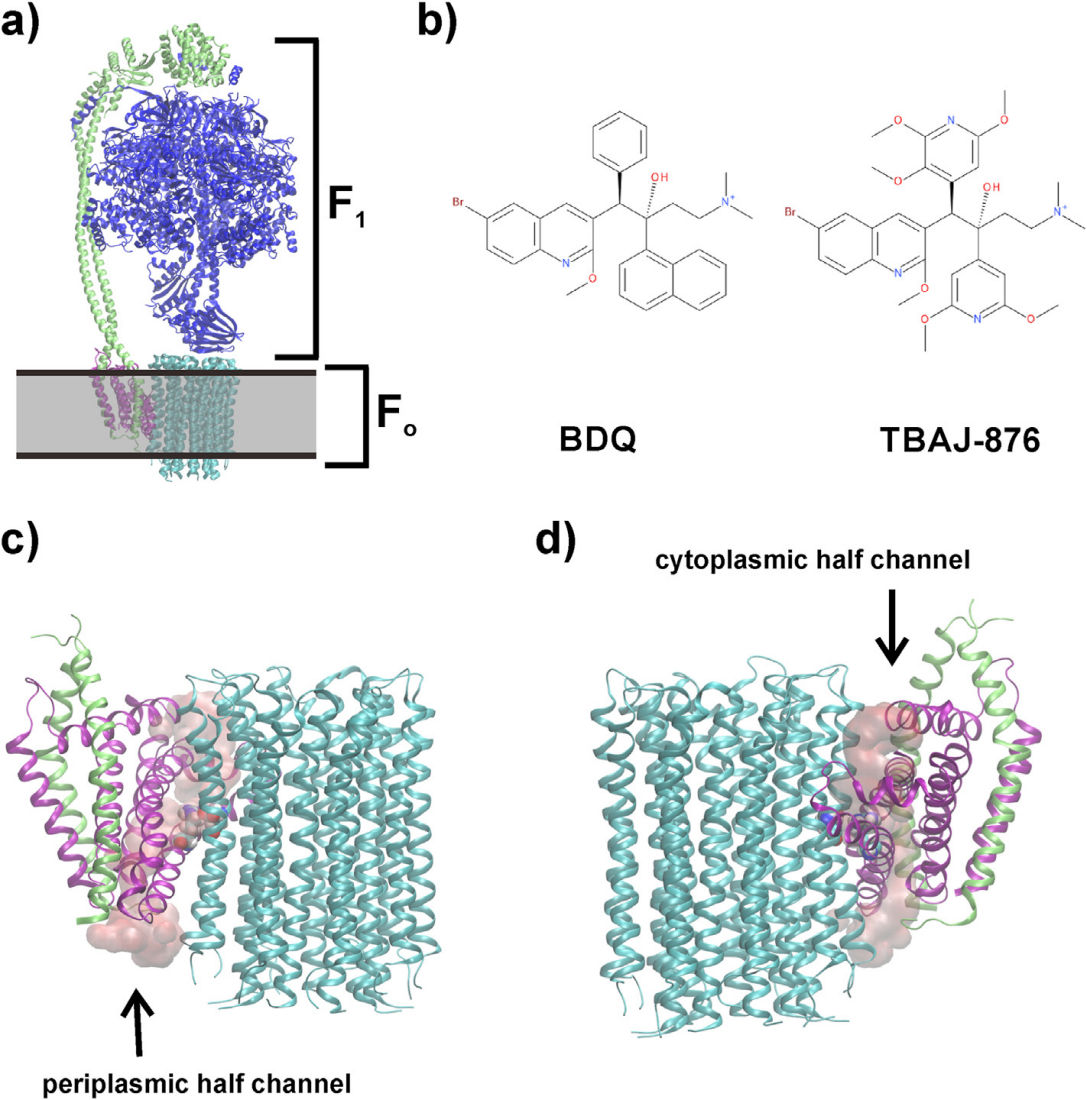
**Mycobacterial F-ATP synthase and two drugs binding to the enzyme.** In a) the whole F-ATP synthase is shown, and the membrane location is schematically indicated in grey. In b) the chemical structures of the drugs BDQ and TBAJ-876 are shown. In c) and d) the periplasmic and cytoplasmic ion-accessible half channels are shown in red surface representation. The *c*-ring is shown in cyan, subunit *a* in purple and the peripheral stalk and subunit δ in lime, and the remaining F_1_ subunits in blue. The channel separating key-arginine and key-glutamate are shown in van der Waals spheres. (For interpretation of the references to color in this figure legend, the reader is referred to the Web version of this article.)

BDQ, approved for clinical use in 2012 (FDA, 2012), is a first-in-class diarylquinoline (DARQ) (Andries et al., 2005) used for the treatment of multidrug-resistant TB. Its bactericidal potency against nonreplicating sub-populations and high efficacy in humans have validated energy metabolism pathways as an attractive target space for drug development (Hards et al., 2015; Koul et al., 2008). The drug targets the F_1_F_O_-ATP synthase (F-ATP synthase), which is essential for growth and viability of the pathogen (McNeil et al., 2020; Saw et al., 2019), and generates ATP in the process of OXPHOS by using the electrochemical gradient generated by the electron transport chain. The mycobacterial F_1_F_O_-ATP synthase consists of nine subunits with a stoichiometry of α_3_:β_3_:γ:δ:ε:*a*:*b*:*b’*:*c*_9_ (Fig. 1) (Guo et al., 2021; Kamariah et al., 2019). The membrane-embedded F_O_ subunit *a* and the rotating *c*-ring, composed of nine *c* subunits forming a helix-loop-helix structure (Guo et al., 2021; Preiss et al., 2015), translocate protons from the intermembrane space to the cytoplasm via two half-channels (Fillingame and Steed, 2014) in subunit *a* (Fig. 1) (Guo et al., 2021; Montgomery et al., 2021); these half-channels are separated by an essential arginine residue in subunit *a* (Mitome et al., 2010), which interacts with an essential, proton-translocating (Sebald et al., 1980) glutamate of the *c*-ring (Fig. 1), causing the protonation change (Kubo et al., 2020; Pogoryelov et al., 2010). The revolution of the *c*-ring also drives rotation of the central stalk subunits γ and ε, which causes conformational changes in the catalytic sites of the α_3_β_3_ hexamer leading to ATP formation (Guo et al., 2021; Montgomery et al., 2021). The peripheral stalk subunits *b*:*b’:*δ provide the flexibility to smoothen the transmission of power between the rotary *c*-ring and the α_3_:β_3_:γ:ε domain (Harikishore et al., 2022; Montgomery et al., 2021).

**Fig. 2:**
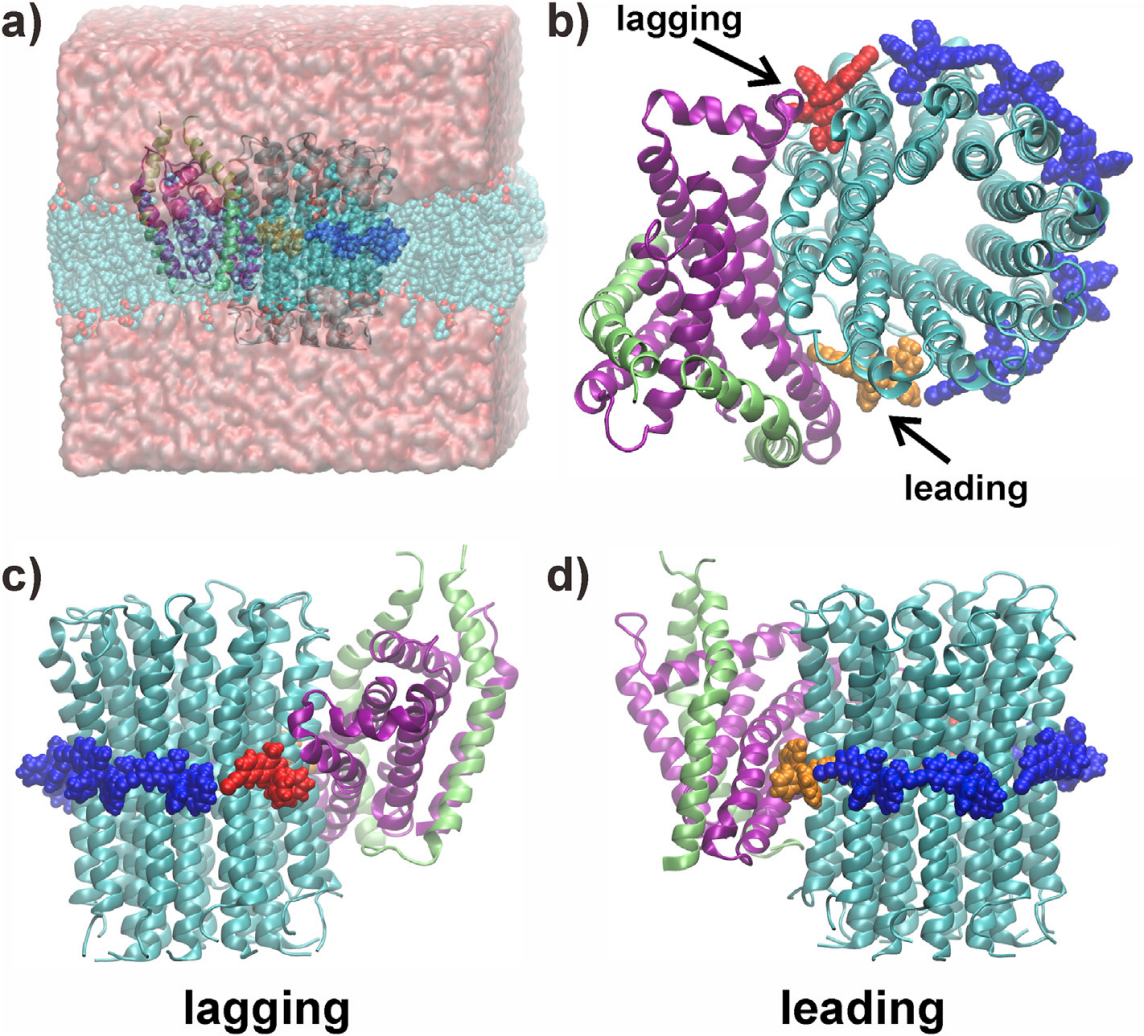
**BDQ binding to the *M. smegmatis* F**_**O**_**domain.** Snapshots are shown of a) the entire membrane-bound system, b) the top-view omitting solvent and lipid, and close-ups of c) leading and d) lagging sites. BDQ molecules bound to the leading, lagging and *c*-ring only sites are colored in *orange*, *red* and *blue*, respectively. Subunits *a*, *c* and *b* are shown in *purple*, *cyan* and *lime*, respectively. In a), lipids and solvent are shown in transparent representation for clarity. (For interpretation of the references to color in this figure legend, the reader is referred to the Web version of this article.)

The cryo-electron microscopy (cryo-EM) structure of the *M. smegmatis* F_1_F_O_-ATP synthase revealed that five BDQ molecules bind with lower affinity to the *c*-ring, with the dimethylamino group of each BDQ interacting with the carboxyl group of the proton-carrying residue E65. Both E65 and BDQ are likely charged, as suggested by biochemical experiments (Haagsma et al., 2011). These experiments were further evaluated by simulations of the mitochondrial F-ATP synthase bound to BDQ, which could show that the charged state is bound stably to the enzyme while the neutral form was unstable (Luo et al., 2020). In addition, two molecules bind with higher affinity to two respective subunit *a*/*c*-interfaces, denoting ‘leading-’ and ‘lagging sites’ (Fig. 2 (Guo et al., 2021)). The leading site involves a *c* subunit that has just interacted with subunit *a* and picked up a proton from the periplasm, while the lagging site involves a *c* subunit poised to interact with subunit *a* to deposit a proton into the cytoplasm (Guo et al., 2021). Therefore, BDQ's wedge-like binding to the two subunit *a*/*c*-interfaces blocks rotation. A second mechanism of action proposed includes BDQ's function as an ionophore that would disturb the transmembrane ion gradient (Hards et al., 2018).

The successful therapeutic advance of BDQ has been overshadowed by the development of clinical resistance (Andries et al., 2014) along with the observations that the drug also binds to the mitochondrial F-ATP synthase (Luo et al., 2020) and the cardiac human ether-a-go-go-related gene (hERG) potassium ion channels (Sutherland et al., 2018). The suppression of the mitochondrial enzyme (Luo et al., 2020) or the hERG channel (Sanguinetti and Tristani-Firouzi, 2006) by BDQ could potentially cause fatal heart arrythmias during treatment of mycobacterial infections. These side effects were reported to cause an increased number of deaths in comparison to the control group (FDA, 2012). Recent medicinal chemistry campaigns resulted in a new generation of DARQ analogs of BDQ that have the potential to address these issues (Sutherland et al., 2019, 2018; Tantry et al., 2017). TBAJ-876, which is currently in phase 1 trials, is a newly developed compound of this series (Fig. 1b), showing improved physio-chemical properties (Sutherland et al., 2019) and is bactericidal (Sarathy et al., 2020b, 2019). To pave the way for further structure-activity relationship studies and novel design of DARQ analogs, structural and energetic information regarding TBAJ-876's binding mechanism are required. Here, we use molecular dynamics (MD) simulations and free energy calculations to describe the structural and energetic basis of BDQ- and TBAJ-876-binding to the *M. smegmatis* F_O_ domain.

## 2 Material & methods

### 2.1 Conventional MD simulations

To generate the input coordinates for simulations, we used the structure of the BDQ bound F_O_ domain of the *M. smegmatis* F-ATP synthase (PDB-ID: 7JGC) (Guo et al., 2021). This protein-drug complex was inserted into a 1-palmitoyl-2-oleoyl-sn-glycero-3-phosphocholine (POPC) membrane bilayer using the CHARMM Graphical User Interface (GUI) (Jo et al., 2008) Membrane Builder module (Wu et al., 2014), as shown in Fig. 2. A ‘lipid plug’ was introduced inside the central cavity of the *c*-ring, as described previously (Krah et al., 2020) and confirmed experimentally (Meier et al., 2001). A physiological NaCl concentration and additional Cl^-^ counter ions were added. Temperature and pressure were kept constant at 300 ​K and 1 ​bar, using the velocity-rescale thermostat (Bussi et al., 2007) and the Parrinello Rahman barostat (Parrinello and Rahman, 1981). We simulated two drugs bound to the F_O_ domain from *M. smegmatis*: BDQ, which is resolved in the respective cryo-EM structure (Guo et al., 2021), and TBAJ-876. For the TBAJ-876 bound state, we conducted a least squares fit of TBAJ-876 over the position of BDQ. After 20 ns of equilibration, restraining backbone atoms, unrestrained simulations were carried out for 100 ns in triplicate for both drugs, for systems in which the drug and key-glutamate were treated as either neutral or charged; only the key-glutamate bound to the essential arginine (R188) (Mitome et al., 2010) dividing both half-channels (Fillingame and Steed, 2014) was set to be charged in all systems. We used the CHARMM36m force field for proteins (Huang et al., 2016), CHARMM36 force field for lipids (Klauda et al., 2010) and the CGENFF force field (Vanommeslaeghe et al., 2010) for the bound drugs. The TIP3P water model (Jorgensen et al., 1983) was used. Simulations were carried out with the GROMACS (v2018) package (Abraham et al., 2015), using an integration time step of 2 fs. Electrostatic interactions were calculated the Particle Mesh Ewald method with a 12 ​Å real space cut-off. Van der Waals contacts were switched after 8 ​Å applying a cut-off of 12 ​Å. The LINCS (Hess et al., 1997) algorithm was used to restrain all bonds involving hydrogen atoms.

### 2.2 Free energy calculations

We calculated the binding free energy of each drug using the thermodynamic integration (TI) approach, when drug and key-glutamate were in the neutral state; this approach was chosen as a deprotonated key-glutamate induces a potentially artefactual water funnel connecting the solvent and the key-glutamate (Gohlke et al., 2012; Krah et al., 2010). Although the drug likely binds in its charged state (Haagsma et al., 2011), assuming that the protonation free energy is similar in each site then water intrusion is unlikely to be observed, making our chosen approach more appropriate. During TI, we changed the coupling parameter λ from 0 (drug fully present) to 1 (drug fully dissolved) over 27 windows. Each window was simulated for 1 ns and the first 100 ps of each were discarded for subsequent analysis using the Bennet Acceptance Ratio (BAR) approach (Bennett, 1976). We applied flat bottom restraints to prevent the drug from dissociation from the binding site. An entropic correction based on the accessible volume that each drug could access within the binding site with respect to the standard volume of water was applied; the accessible volume (Wang et al., 2006) was calculated with *trj_cavity* (Paramo et al., 2014) and subtracted from the volume of the drug calculated as described previously (Zhao et al., 2003). We repeated each TI calculation using three independent sets of simulations. The output coordinates of the conventional MD simulations were used as the starting structure.

## 3 Results & discussion

### 3.1 A different interaction network of each drug highlights their different affinities

We first simulated the drugs BDQ and TBAJ-876 bound to the F_O_ domain in two states. First, the drug and key-glutamate (E65) were simulated in their charged states and second in their neutral states. Irrespective of the protonation state of E65/drugs, we observed a comparable number of hydrogen bonds and hydrophobic contacts between drug and protein (cut-off 4 ​Å) when comparing the same binding site (Table 1). However, it has previously been postulated that BDQ and the key-glutamate are charged (Haagsma et al., 2011). For either drug, the leading site exhibited the highest number of hydrophobic contacts, followed by the lagging site, and finally the *c*-ring only site with the least number of hydrophobic contacts. When comparing between the two drugs, a significant increase in hydrophobic contacts was observed for TBAJ-876 compared to BDQ (Table 1). The higher affinity of TBAJ-876 compared to BDQ (Sarathy et al., 2019) could be caused by these additional hydrophobic interactions.

**Table 1:** **Number of hydrogen bonds and heavy-atom contacts between each drug and the protein (cut-off 4 ​Å).** Average and standard deviations for each system were obtained over the whole triplicate simulation sampling (300 ns). The number of contacts using a larger cut-off distance of 5 ​Å is shown in Table S1, and follow the same trend.

a) drug positively and E65 negatively charged					
Number of hydrogen bonds			Number of contacts		
	BDQ	TBAJ-876		BDQ	TBAJ-876
Lagging	1.4 ​± ​0.6	1.4 ​± ​0.5	**Lagging**	26.3 ​± ​4.2	32.2 ​± ​4.0
Leading	1.2 ​± ​0.5	1.1 ​± ​0.4	**Leading**	31.4 ​± ​4.9	35.4 ​± ​4.7
*c*-ring	1.2 ​± ​0.5	1.3 ​± ​0.5	***c*-ring**	22.1 ​± ​3.5	23.0 ​± ​3.3
b) drug and E65 neutral					
Number of hydrogen bonds			Number of contacts		
	BDQ	TBAJ-876		BDQ	TBAJ-876
Lagging	1.0 ​± ​0.2	0.9 ​± ​0.3	**Lagging**	25.0 ​± ​3.8	28.1 ​± ​3.9
Leading	0.9 ​± ​0.3	1.0 ​± ​0.3	**Leading**	32.1 ​± ​3.8	32.7 ​± ​5.5
*c*-ring	0.8 ​± ​0.4	0.8 ​± ​0.4	***c*-ring**	19.2 ​± ​3.4	19.8 ​± ​3.9

To assess binding mode of each drug to the F_O_ domain, we measured the minimum distance between residues in close contact with the drug, for both protonation states simulated (Table 2). While the binding contacts at the *c*-ring only site were similar for both drugs, some variations in interacting residues were observed at the leading and lagging sites, which may promote stronger binding for a given drug. The minimum distances for both drugs with the protein in the charged state are shown in Table 2, the minimum distances for the neutral state are shown in Table S2.

**Fig. 3:**
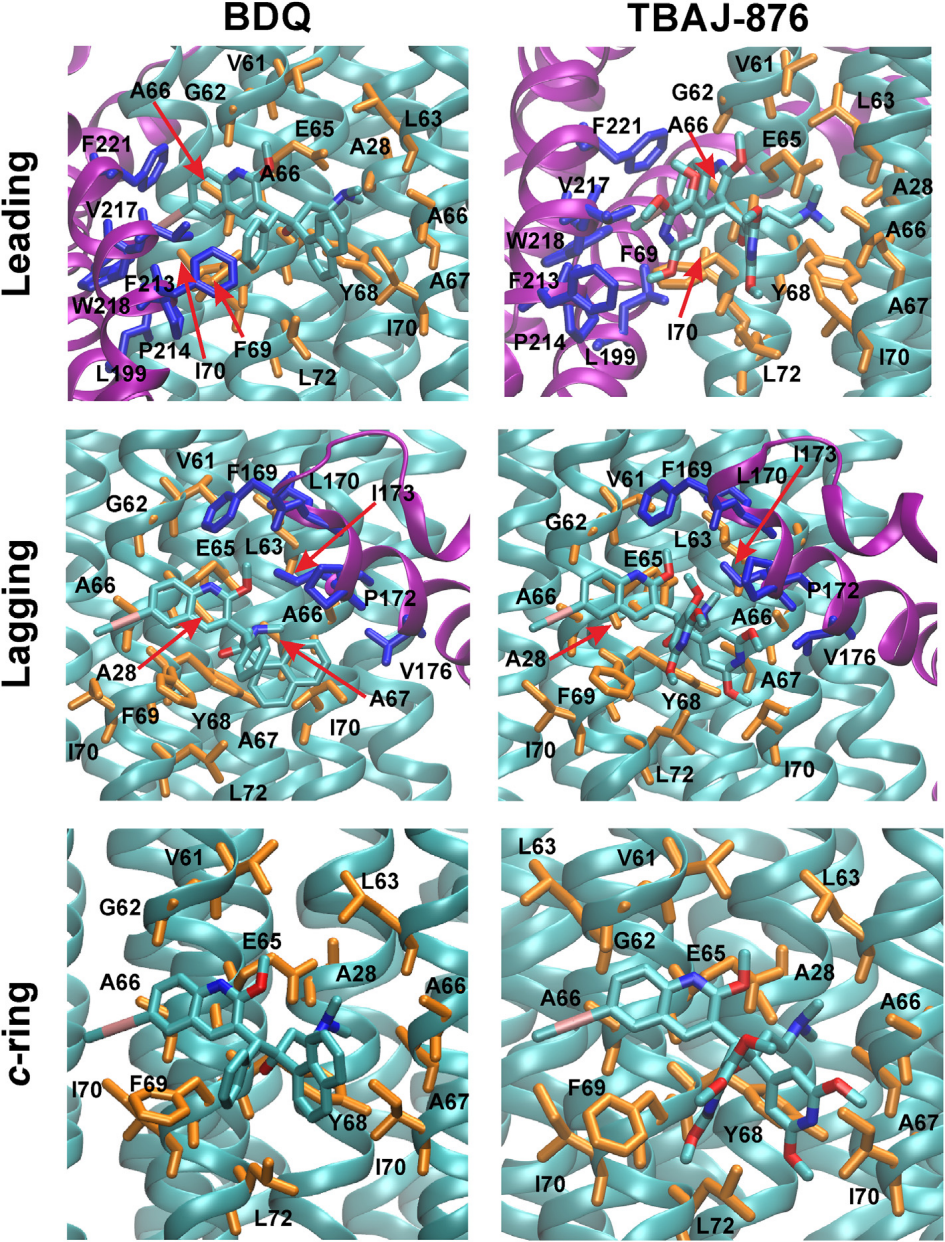
**Binding of the drug at each site.** Molecular representations are shown for each drug bound to the leading, lagging and *c*-ring only sites when being charged, as indicated by corresponding labels for each panel. Residues from the *c*-ring and subunit *a* are shown in orange and blue, respectively. Figures were prepared by using VMD (Humphrey et al., 1996). Corresponding LigPlot+ (Laskowski and Swindells, 2011) representations for each bound state are shown in Fig. S1. (For interpretation of the references to color in this figure legend, the reader is referred to the Web version of this article.)

Both drugs are coordinated in the *c*-ring only site by a salt bridge between E65:Oεx and drug:N^+^ groups, along with hydrophobic contacts of different strength and stability formed between each drug and *c*A28, *c*V61, *c*G62, *c*L63, *c*E65, *c*A66, *c*A67, *c*Y68, *c*F69, *c*I70 and *c*L72. BDQ and TBAJ-876 are additionally coordinated in the lagging site via non-polar contacts with *a*L170, *a*P172, *a*I173 and *a*V176. In addition, *c*F74 may coordinate the drugs at the lagging site, though this needs further experimental evaluation as the data are not conclusive (Tables 2 and S3). For all simulations of TBAJ-876 and the majority of simulations of BDQ at the lagging site, *a*F169 also coordinates the drug, especially in the latter stages of the simulations (Table S3), indicating that *a*F169 is also potentially part of the BDQ binding site. In addition to residues in the *c*-ring only site, both drugs at the leading site are stabilized by contacts with *a*F213, *a*P214, *a*V217, *a*W218 and *a*F221. Furthermore, *a*L199 coordinates TBAJ-876 and possibly also BDQ (Tables 2 and S3). In both the leading and lagging sites, the average minimum distances of protein residues with the drugs were in general observed to be slightly decreased in the TBAJ-876- compared to the BDQ-bound state, although the distributions overlap. The binding sites of both drugs are depicted in Figs. 3 and S1. The additional coordinating residues, associated with tighter coordination and greater number of hydrophobic contacts (Table 1) for TBAJ-876 help to rationalize its lower experimentally determined IC_50_ compared to BDQ (Sarathy et al., 2019). The coordination pattern in the neutral state is similar to those described for the charged state (Table S2).

**Table 2:** **Minimum distance of protein residues to BDQ or TBAJ-876.** Distances are shown for both drugs bound to each site (leading, lagging and *c*-ring only) when drug and key-glutamate are charged. Distances are reported in Å. Average and standard deviations for each system were obtained over the whole triplicate simulation sampling (300 ns).

	BDQ (deprotonated)			TBAJ-876 (deprotonated)		
	*c*-ring	Lagging	Leading	*c*-ring	Lagging	Leading
*c*A28	5.1 ​± ​0.6	4.9 ​± ​0.6	5.4 ​± ​0.7	5.0 ​± ​0.5	5.0 ​± ​0.5	5.2 ​± ​0.7
*c*V61	4.8 ​± ​0.6	5.3 ​± ​0.2	5.2 ​± ​0.9	4.9 ​± ​0.6	4.6 ​± ​0.5	5.0 ​± ​0.9
*c*G62	4.1 ​± ​0.4	4.4 ​± ​0.4	4.4 ​± ​0.4	4.1 ​± ​0.3	4.0 ​± ​0.2	4.5 ​± ​0.4
*c*L63	4.0 ​± ​0.4	4.4 ​± ​0.6	4.2 ​± ​0.6	4.0 ​± ​0.3	4.0 ​± ​0.3	4.4 ​± ​0.8
*c*E65	3.5 ​± ​0.1	3.5 ​± ​0.2	3.5 ​± ​0.2	3.5 ​± ​0.1	3.5 ​± ​0.1	3.5 ​± ​0.2
*c*A66	3.6 ​± ​0.2	3.6 ​± ​0.2	3.6 ​± ​0.2	3.6 ​± ​0.2	3.6 ​± ​0.2	3.6 ​± ​0.2
*c*A67	3.8 ​± ​0.2	3.7 ​± ​0.2	4.0 ​± ​0.3	3.9 ​± ​0.3	3.7 ​± ​0.2	3.9 ​± ​0.3
*c*Y68	3.8 ​± ​0.2	3.7 ​± ​0.2	3.8 ​± ​0.2	3.8 ​± ​0.2	3.8 ​± ​0.2	3.8 ​± ​0.2
*c*F69	3.6 ​± ​0.2	3.6 ​± ​0.2	3.6 ​± ​0.2	3.4 ​± ​0.2	3.4 ​± ​0.2	3.5 ​± ​0.2
*c*I70	3.7 ​± ​0.3	3.6 ​± ​0.2	3.7 ​± ​0.4	3.6 ​± ​0.2	3.6 ​± ​0.2	3.6 ​± ​0.2
*c*L72	5.0 ​± ​1.3	3.9 ​± ​0.3	4.8 ​± ​1.0	3.8 ​± ​0.8	3.5 ​± ​0.3	4.5 ​± ​0.9
*c*F74	7.1 ​± ​1.2	5.3 ​± ​0.7	7.1 ​± ​1.1	5.5 ​± ​1.2	5.1 ​± ​1.0	6.9 ​± ​2.0
*c*E65:Oεx-LIG:N^+^	2.7 ​± ​0.1	2.7 ​± ​0.1	2.7 ​± ​0.1	2.7 ​± ​0.1	2.7 ​± ​0.1	2.7 ​± ​0.1
*a*F169	N/A	5.6 ​± ​1.5	N/A	N/A	4.5 ​± ​0.9	N/A
*a*L170	N/A	4.3 ​± ​0.5	N/A	N/A	4.0 ​± ​0.4	N/A
*a*P172	N/A	3.9 ​± ​0.3	N/A	N/A	3.6 ​± ​0.3	N/A
*a*I173	N/A	3.6 ​± ​0.2	N/A	N/A	3.5 ​± ​0.2	N/A
*a*V176	N/A	3.8 ​± ​0.3	N/A	N/A	3.8 ​± ​0.3	N/A
*a*L199	N/A	N/A	5.3 ​± ​1.0	N/A	N/A	4.9 ​± ​1.2
*a*F213	N/A	N/A	4.1 ​± ​0.5	N/A	N/A	3.8 ​± ​0.5
*a*P214	N/A	N/A	5.5 ​± ​1.0	N/A	N/A	4.3 ​± ​0.6
*a*V217	N/A	N/A	3.8 ​± ​0.3	N/A	N/A	3.8 ​± ​0.4
*a*W218	N/A	N/A	2.2 ​± ​0.4	N/A	N/A	2.4 ​± ​0.5
*a*F221	N/A	N/A	3.3 ​± ​0.4	N/A	N/A	3.2 ​± ​0.4

### 3.2 The lagging site is the high affinity binding site

**Fig. 4:**
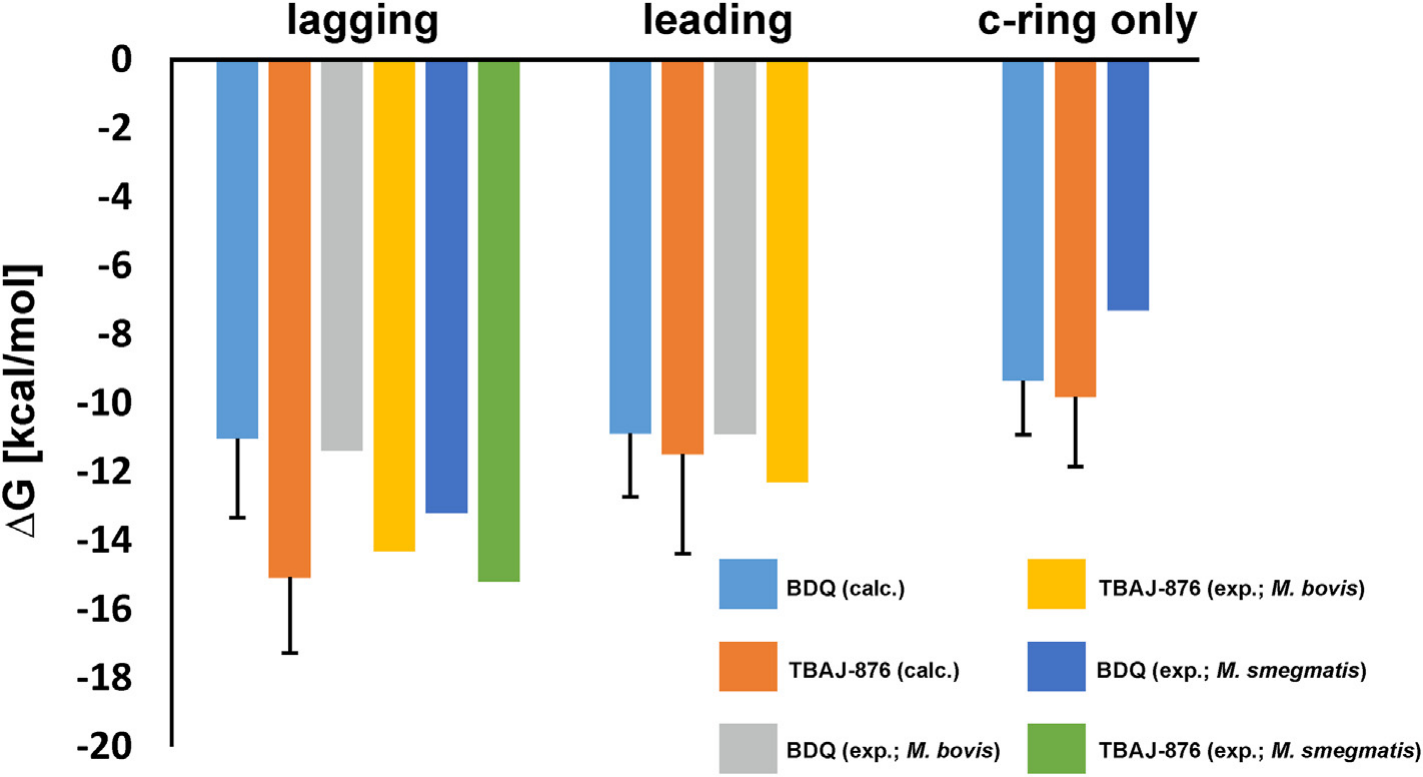
**Binding free energy calculations for BDQ and TBAJ-876 to the *M. smegmatis* F**_**O**_**domain.** Calculated free energy values are shown in cyan and orange for BDQ for TBAJ-876, respectively. The experimental values (Sarathy et al., 2019) for BDQ and TBAJ-876 bound to the F-ATP synthase of *M. bovis* are shown in grey and yellow, respectively. Experimental data for BDQ binding to the *c*-ring (Haagsma et al., 2011) and both drugs binding to the lagging site of *M. smegmatis* (Haagsma et al., 2011; Sarathy et al., 2019) are shown in dark blue and green, respectively. Experimental data not shown for BDQ and TBAJ-876 binding to the mycobacterial F-ATP synthase are not available, to our knowledge. For the calculated free energies, standard deviations were derived from three independent simulations. (For interpretation of the references to color in this figure legend, the reader is referred to the Web version of this article.)

To estimate which of the three drug bound states is likely to be the high affinity site, we calculated the binding free energy of BDQ/TBAJ-876 binding to the F_O_ domain from *M. smegmatis*. We used the neutral state (E65 protonated/drug not protonated at its amine group) to derive the free energy estimate, as the charged state induces a potentially artefactual water funnel connecting the solvent and the key-glutamate in the absence of ligands (Gohlke et al., 2012; Krah et al., 2010); this state is reproduced if the electrostatic interactions are switched off in the free energy calculations. We find that the *c*-ring only site has the lowest affinity for both drugs. The lagging site, which suppresses the rotation in synthesis direction, has the highest affinity for TBAJ-876, followed by the leading site. The free energy difference between the leading and lagging site in the BDQ bound state is very low (ΔΔG (lagging → leading) ​= ​0.1 ​kcal/mol) and within the standard deviation. This is in agreement with experiments (Sarathy et al., 2019) which showed only a two-fold decreased IC_50_ inhibition in the synthesis direction for BDQ. These results (Fig. 4) confirm that subunit *a* contributes remarkably to the binding of the drugs and is likely responsible for the higher affinity compared to the affinity of the drug solely bound to the *c*-ring, in agreement with experiment (Guo et al., 2021; Sarathy et al., 2019).

Overall, the difference in predicted relative binding free energies comparing BDQ and TBAJ-876 match up with the trend in measured affinities. While there is no experimental data available for the *c*-ring only site for TBAJ-876, the ΔΔG vales for the leading and lagging sites were predicted to be 1.8 ​kcal/mol and 5.2 ​kcal/mol, compared to experimental measures of 1.4 ​kcal/mol and 2.9 ​kcal/mol for *M. bovis*, respectively (Haagsma et al., 2011; Sarathy et al., 2019); the IC_50_ for this organism is decreased by only an order of magnitude in the ATP synthesis direction compared to *M. smegmatis* (Sarathy et al., 2019) and thus is a reasonable model to compare to. Within the error range of our calculations, our predictions thus rationalize TBAJ-876's improved affinity at both sites.

### 3.3 Mechanistic implications

**Fig. 5:**
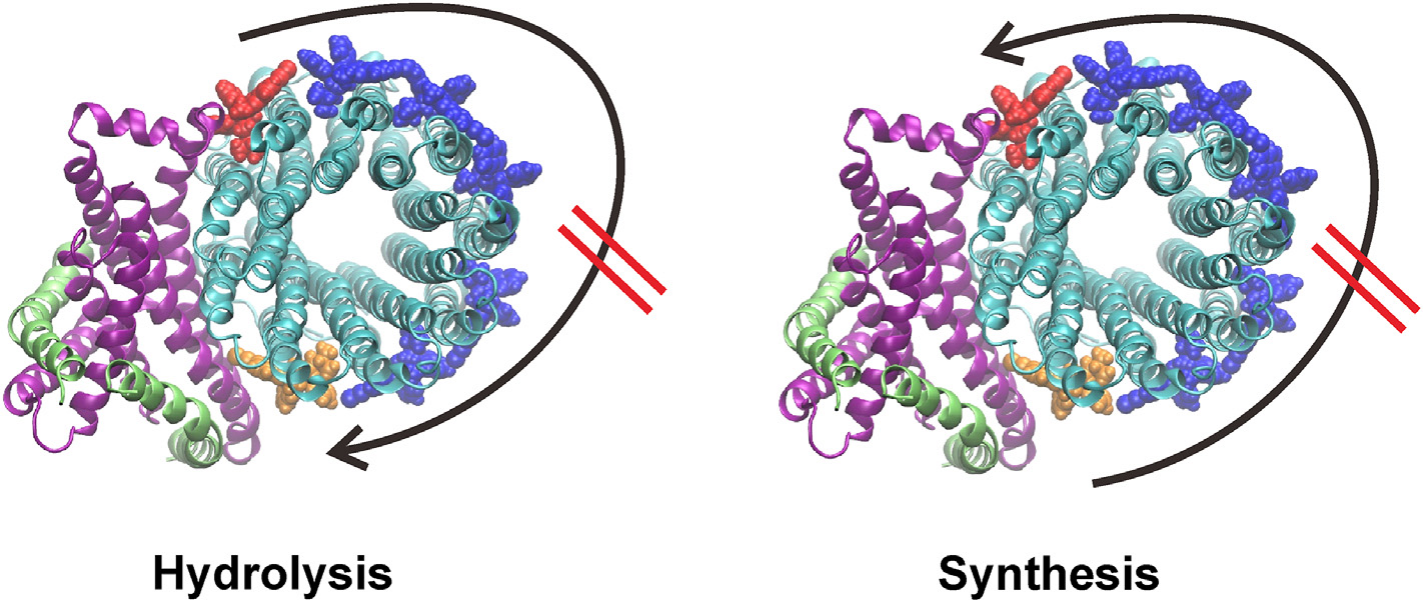
**Inhibition of rotary mechanism by drugs bound to the F**_**O**_**domain.** BDQ and TBAJ-876 bind preferably at the leading or lagging sites, inhibiting the rotation of the *c*-ring via steric clashes with subunit *a* in hydrolysis or synthesis directions, respectively. BDQ molecules bound to the leading, lagging and *c*-ring only sites are colored in *orange*, *red* and *blue*, respectively. Subunits *a*, *c* and *b* are shown in *purple*, *cyan* and *lime*, respectively. (For interpretation of the references to color in this figure legend, the reader is referred to the Web version of this article.)

Our simulations show that both drugs have higher affinity for binding to the leading and lagging sites compared to the *c*-ring only site. These calculations are in agreement with experimental data, which showed that: 1) the affinity measured for the *c*-ring is lower than for the whole enzyme (Haagsma et al., 2011); and 2) that BDQ bound to the *c*-ring, but not subunit *a*, could be washed away, whereas drugs at the leading and lagging sites maintained their presence (Guo et al., 2021). Our calculations and the experimental data indicate that the drugs become bound at the leading and lagging site with different affinities, which also influences the inhibition of function in the respective synthesis and hydrolysis directions (Sarathy et al., 2019). Being bound at the *a*/*c*-interface, the drugs likely suppress rotation of the *c*-ring via steric clashes with subunit *a*. This inhibited revolution thereby prevents proton translocation via the half-channels in subunit *a* (Fig. 5).

## 4 Conclusions

We have elucidated the binding sites of the drugs BDQ and TBAJ-876 bound to the F_O_ domain from *M. smegmatis*, resolved the energetic basis for drug binding, and finally discussed this in the context of how the drugs likely inhibit the enzyme function. This represents a framework which may potentially be used to predict novel lead compounds that bind to other pathogenic bacterial F-ATP synthases essential for their survival (Vestergaard et al., 2022), such as e.g. the ESKAPE organism *Acinetobacter baumannii*, whose structure was recently solved (Demmer et al., 2022). The data presented also provides a platform for future studies on TBAJ-876's potency for *M. abscessus*, the causative agent of pulmonary disease. The drug displays attractive *in vitro* and *in vivo* activities against the *Mab* complex strains, although with lower potency and reduced bactericidal activity, like BDQ (Sarathy et al., 2020a).

## CRediT authorship contribution statement

**Alexander Krah:** Conceptualization, Formal analysis, Investigation, Visualization, Writing – original draft, Writing – review & editing. **Gerhard Grüber:** Writing – original draft, Writing – review & editing. **Peter J. Bond:** Formal analysis, Supervision, Funding acquisition, Writing – review & editing.

## Declaration of competing interest

The authors declare that they have no known competing financial interests or personal relationships that could have appeared to influence the work reported in this paper.
