# Supplementary material for: Binding properties of the anti-TB drugs bedaquiline and TBAJ-876 to a mycobacterial F-ATP synthase
Source: Curr Res Struct Biol. 2022 Sep 9;4:278–84. doi: 10.1016/j.crstbi.2022.09.001 (PMC9516385; doi:10.1016/j.crstbi.2022.09.001)
Supplement: Multimedia component 1 [file mmc1.pdf]

Supplementary Information

Binding properties of the anti-TB drugs bedaquiline and TBAJ-876 to a mycobacterial F-ATP synthase

Alexander Krah[1*], Gerhard Grüber[1,2] & Peter J. Bond[1,3*]

[1]Bioinformatics Institute, Agency for Science, Technology and Research (A*STAR), 30 Biopolis Str., #07-01 Matrix, Singapore 138671

[2]School of Biological Sciences, Nanyang Technological University, 60 Nanyang Drive, Singapore 637551

[3]Department of Biological Sciences, National University of Singapore, 14 Science Drive 4, Singapore 117543

Contact: kraha@bii.a-star.edu.sg (AK) / peterjb@bii.a-star.edu.sg (PJB)

Table S1: Number of heavy-atom contacts between each drug and the protein (cut-off 5 Å). Average and standard deviations for each system were obtained over the whole triplicate simulation sampling (300 ns).

**a) drug and E65 charged**		
**Number of contacts**		
	**BDQ**	**TBAJ-876**
**Lagging**	64.4 ± 4.1	72.5 ± 4.2
**Leading**	74.4 ± 5.6	81.2 ± 6.4
***c*-ring**	50.7 ± 5.1	52.5 ± 4.3

**b) drug and E65 neutral**		
**Number of contacts**		
	**BDQ**	**TBAJ-876**
**Lagging**	60.7 ± 4.3	65.5 ± 4.6
**Leading**	74.6 ± 5.5	76.6 ± 10.2
***c*-ring**	44.8 ± 4.5	45.7 ± 5.3

Table S2: Minimum distances of protein residues to BDQ and TBAJ-876. Distances are shown for both drugs bound to each site (leading, lagging and *c*-ring only) when drug and key-glutamate are neutral. Averages and standard deviations were derived from merging all data over all simulated trajectories (300 ns each).

	BDQ (protonated)			TBAJ-876 (protonated)		
	c-ring	Lagging	Leading	*c*-ring	Lagging	Leading
A28	5.8 ± 0.9	4.7 ± 0.5	5.4 ± 0.5	5.7 ± 1.1	5.0 ± 0.5	5.1 ± 0.6
V61	5.2 ± 0.7	5.7 ± 0.5	5.4 ± 0.5	5.4 ± 0.7	5.2 ± 0.5	5.6 ± 0.5
G62	4.4 ± 0.4	4.5 ± 0.4	4.4 ± 0.3	4.4 ± 0.4	4.2 ± 0.3	4.4 ± 0.4
L63	4.3 ± 0.6	4.6 ± 0.5	4.3 ± 0.5	4.4 ± 0.6	4.5 ± 0.5	4.4 ± 0.6
E65	3.6 ± 0.2	3.6 ± 0.2	3.6 ± 0.2	3.6 ± 0.3	3.6 ± 0.2	3.6 ± 0.3
A66	3.6 ± 0.2	3.6 ± 0.2	3.6 ± 0.2	3.6 ± 0.3	3.6 ± 0.2	3.6 ± 0.2
A67	4.4 ± 1.0	3.8 ± 0.2	3.8 ± 0.2	4.2 ± 1.1	3.8 ± 0.2	3.9 ± 0.3
Y68	3.7 ± 0.3	3.6 ± 0.2	3.8 ± 0.3	4.0 ± 1.0	3.6 ± 0.2	3.7 ± 0.2
F69	3.5 ± 0.2	3.6 ± 0.2	3.6 ± 0.2	3.5 ± 0.1	3.4 ± 0.2	3.4 ± 0.2
I70	3.7 ± 0.3	3.7 ± 0.2	3.6 ± 0.2	3.8 ± 0.2	3.7 ± 0.2	3.6 ± 0.2
L72	4.6 ± 1.1	3.8 ± 0.3	4.5 ± 0.6	4.6 ± 1.7	3.8 ± 0.4	3.8 ± 0.6
F74	6.8 ± 1.2	5.0 ± 0.8	7.2 ± 1.2	6.2 ± 2.3	4.3 ± 0.7	4.9 ± 1.1
**E65:Oε2 - LIG:N^{neutral}**	3.5 ± 1.3	2.8 ± 0.1	2.8 ± 0.2	3.2 ± 0.2	2.8 ± 0.1	2.8 ± 0.2
F169	N/A	5.17 ± 1.14	N/A	N/A	4.5 ± 0.8	N/A
L170	N/A	4.41 ± 0.54	N/A	N/A	4.0 ± 0.4	N/A
P172	N/A	3.98 ± 0.31	N/A	N/A	3.6 ± 0.3	N/A
I173	N/A	3.62 ± 0.18	N/A	N/A	3.6 ± 0.2	N/A
V176	N/A	3.68 ± 0.29	N/A	N/A	3.8 ± 0.3	N/A
L199	N/A	N/A	5.7 ± 0.7	N/A	N/A	6.3 ± 2.0
F206	N/A	N/A	8.3 ± 1.2	N/A	N/A	5.6 ± 2.4
F213	N/A	N/A	3.8 ± 0.3	N/A	N/A	3.9 ± 0.5
P214	N/A	N/A	4.8 ± 0.7	N/A	N/A	4.6 ± 1.2
V217	N/A	N/A	3.7 ± 0.3	N/A	N/A	4.2 ± 0.8
W218	N/A	N/A	2.3 ± 0.5	N/A	N/A	3.6 ± 1.9
F221	N/A	N/A	2.9 ± 0.4	N/A	N/A	2.6 ± 0.6

Table S3: Block analysis of the minimum distances for both drugs. The minimum distances for windows of 10 – 70 ns, 40 – 70 ns and 70 – 100 ns are shown for all three sites. In a) and b) the BDQ bound state is shown in its charged and neutral state, respectively. In c) and d) the minimum distances for the TBAJ-876 bound state are shown when drug and key-glutamate are charged or neutral, respectively.

**a) BDQ (deprotonated)**	***c*-ring**			**Lagging**			**Leading**		
	**10-40 ns**	**40-70 ns**	**70-100 ns**	**10-40 ns**	**40-70 ns**	**70-100 ns**	**10-40 ns**	**40-70 ns**	**70-100 ns**
***c*A28**	4.9 ± 0.6	4.8 ± 0.6	4.7 ± 0.6	4.9 ± 0.6	4.8 ± 0.5	4.8 ± 0.6	5.4 ± 0.6	5.2 ± 0.7	5.6 ± 0.8
***c*V61**	4.2 ± 0.3	4.2 ± 0.4	4.1 ± 0.5	5.0 ± 0.5	5.4 ± 0.7	5.5 ± 0.7	4.8 ± 0.6	5.3 ± 0.8	5.5 ± 1.0
***c*G62**	4.0 ± 0.4	4.0 ± 0.4	3.9 ± 0.3	4.2 ± 0.3	4.4 ± 0.4	4.5 ± 0.5	4.4 ± 0.3	4.4 ± 0.4	4.6 ± 0.6
***c*L63**	3.5 ± 0.1	3.5 ± 0.1	3.5 ± 0.1	4.2 ± 0.3	4.3 ± 0.4	4.8 ± 0.7	4.0 ± 0.3	4.2 ± 0.4	4.4 ± 0.8
***c*E65**	3.6 ± 0.2	3.6 ± 0.2	3.6 ± 0.3	3.5 ± 0.1	3.5 ± 0.1	3.6 ± 0.2	3.5 ± 0.1	3.5 ± 0.1	3.6 ± 0.2
***c*A66**	3.8 ± 0.2	3.8 ± 0.2	3.8 ± 0.2	3.6 ± 0.2	3.6 ± 0.2	3.6 ± 0.3	3.6 ± 0.2	3.6 ± 0.2	3.6 ± 0.2
***c*A67**	3.8 ± 0.2	3.8 ± 0.2	3.8 ± 0.2	3.7 ± 0.3	3.7 ± 0.2	3.8 ± 0.2	3.8 ± 0.2	3.9 ± 0.3	4.2 ± 0.4
***c*Y68**	3.6 ± 0.2	3.6 ± 0.2	3.6 ± 0.2	3.7 ± 0.1	3.7 ± 0.2	3.7 ± 0.2	3.8 ± 0.2	3.8 ± 0.2	3.7 ± 0.2
***c*F69**	3.7 ± 0.3	3.7 ± 0.3	3.7 ± 0.3	3.6 ± 0.2	3.6 ± 0.2	3.6 ± 0.2	3.6 ± 0.2	3.6 ± 0.2	3.6 ± 0.2
***c*I70**	5.0 ± 1.2	5.1 ± 1.3	5.2 ± 1.4	3.6 ± 0.2	3.6 ± 0.2	3.6 ± 0.2	3.6 ± 0.2	3.6 ± 0.2	3.9 ± 0.5
***c*L72**	7.1 ± 1.2	7.1 ± 1.2	7.1 ± 1.3	3.9 ± 0.3	3.8 ± 0.3	3.6 ± 0.2	4.7 ± 0.7	4.5 ± 0.7	5.1 ± 1.5
***c*F74**	2.7 ± 0.1	2.7 ± 0.1	2.7 ± 0.1	5.5 ± 0.6	5.0 ± 0.7	5.0 ± 0.7	7.1 ± 0.8	6.9 ± 0.9	7.1 ± 1.5
***c*E65:Oεx-LIG:N**	4.9 ± 0.6	4.8 ± 0.6	4.7 ± 0.6	2.7 ± 0.1	2.7 ± 0.1	2.7 ± 0.1	2.7 ± 0.1	2.7 ± 0.1	2.8 ± 0.1
***a*F169**	N/A	N/A	N/A	5.9 ± 1.5	5.6 ± 1.4	5.1 ± 1.4	N/A	N/A	N/A
***a*L170**	N/A	N/A	N/A	4.3 ± 0.5	4.3 ± 0.5	4.5 ± 0.6	N/A	N/A	N/A
***a*P172**	N/A	N/A	N/A	3.9 ± 0.3	3.9 ± 0.3	3.9 ± 0.3	N/A	N/A	N/A
***a*I173**	N/A	N/A	N/A	3.7 ± 0.2	3.6 ± 0.2	3.7 ± 0.2	N/A	N/A	N/A
***a*V176**	N/A	N/A	N/A	3.8 ± 0.3	3.8 ± 0.3	3.7 ± 0.3	N/A	N/A	N/A
***a*L199**	N/A	N/A	N/A	N/A	N/A	N/A	4.9 ± 0.8	5.4 ± 0.9	5.8 ± 1.1
***a*F213**	N/A	N/A	N/A	N/A	N/A	N/A	4.0 ± 0.4	4.1 ± 0.5	4.1 ± 0.6
***a*P214**	N/A	N/A	N/A	N/A	N/A	N/A	5.4 ± 0.6	5.2 ± 0.7	6.0 ± 1.2
***a*V217**	N/A	N/A	N/A	N/A	N/A	N/A	3.8 ± 0.3	3.8 ± 0.3	3.9 ± 0.3
***a*W218**	N/A	N/A	N/A	N/A	N/A	N/A	2.1 ± 0.3	2.2 ± 0.5	2.4 ± 0.5
***a*F221**	N/A	N/A	N/A	N/A	N/A	N/A	3.2 ± 0.4	3.3 ± 0.4	3.5 ± 0.5

b) BDQ (protonated)	*c*-ring			Lagging			Leading		
	10-40 ns	40-70 ns	70-100 ns	10-40 ns	40-70 ns	70-100 ns	10-40 ns	40-70 ns	70-100 ns
***c*A28**	5.7 ± 0.8	6.0 ± 0.9	6.0 ± 0.9	4.6 ± 0.5	4.8 ± 0.5	4.9 ± 0.5	5.4 ± 0.5	5.4 ± 0.5	5.5 ± 0.5
***c*V61**	5.4 ± 0.7	5.1 ± 0.8	5.1 ± 0.8	5.7 ± 0.5	5.6 ± 0.5	5.8 ± 0.5	5.4 ± 0.5	5.4 ± 0.5	5.3 ± 0.5
***c*G62**	4.4 ± 0.4	4.3 ± 0.4	4.3 ± 0.4	4.5 ± 0.4	4.4 ± 0.3	4.5 ± 0.4	4.4 ± 0.3	4.4 ± 0.3	4.4 ± 0.3
***c*L63**	4.3 ± 0.5	4.4 ± 0.7	4.4 ± 0.7	4.5 ± 0.5	4.6 ± 0.6	4.6 ± 0.6	4.2 ± 0.4	4.3 ± 0.4	4.3 ± 0.5
***c*E65**	3.6 ± 0.2	3.6 ± 0.2	3.6 ± 0.2	3.6 ± 0.2	3.6 ± 0.2	3.6 ± 0.2	3.6 ± 0.2	3.6 ± 0.2	3.6 ± 0.2
***c*A66**	3.6 ± 0.2	3.6 ± 0.2	3.6 ± 0.2	3.6 ± 0.2	3.6 ± 0.2	3.6 ± 0.2	3.6 ± 0.2	3.6 ± 0.2	3.6 ± 0.2
***c*A67**	4.1 ± 0.8	4.6 ± 1.2	4.6 ± 1.2	3.8 ± 0.2	3.8 ± 0.2	3.8 ± 0.2	3.9 ± 0.3	3.9 ± 0.2	3.8 ± 0.3
***c*Y68**	3.8 ± 0.3	3.7 ± 0.3	3.7 ± 0.3	3.6 ± 0.2	3.7 ± 0.2	3.7 ± 0.2	3.7 ± 0.3	3.8 ± 0.3	3.8 ± 0.3
***c*F69**	3.5 ± 0.2	3.5 ± 0.2	3.5 ± 0.2	3.6 ± 0.2	3.6 ± 0.2	3.6 ± 0.2	3.6 ± 0.2	3.6 ± 0.2	3.5 ± 0.2
***c*I70**	3.6 ± 0.3	3.7 ± 0.3	3.7 ± 0.3	3.7 ± 0.2	3.7 ± 0.2	3.7 ± 0.2	3.6 ± 0.2	3.6 ± 0.2	3.7 ± 0.2
***c*L72**	4.5 1.0	4.7 ± 1.2	4.7 ± 1.2	3.8 ± 0.2	3.8 ± 0.3	3.9 ± 0.3	4.5 ± 0.6	4.4 ± 0.6	4.6 ± 0.7
***c*F74**	6.4 ± 1.1	7.1 ± 1.2	7.1 ± 1.2	4.9 ± 0.8	4.9 ± 0.8	5.2 ± 0.8	6.9 ± 1.0	7.4 ± 1.1	7.8 ± 1.3
***c*E65:Oεx-LIG:N**	3.2 ± 1.0	3.8 ± 1.5	3.9 ± 1.6	2.8 ± 0.1	2.8 ± 0.1	2.8 ± 0.1	2.8 ± 0.1	2.9 ± 0.4	2.8 ± 0.1
***a*F169**	N/A	N/A	N/A	5.6 ± 1.2	5.5 ± 1.2	4.3 ± 0.6	N/A	N/A	N/A
***a*L170**	N/A	N/A	N/A	4.3 ± 0.5	4.5 ± 0.5	4.5 ± 0.6	N/A	N/A	N/A
***a*P172**	N/A	N/A	N/A	3.9 ± 0.3	4.0 ± 0.3	4.1 ± 0.3	N/A	N/A	N/A
***a*I173**	N/A	N/A	N/A	3.6 ± 0.2	3.6 ± 0.2	3.7 ± 0.2	N/A	N/A	N/A
***a*V176**	N/A	N/A	N/A	3.8 ± 0.3	3.7 ± 0.3	3.6 ± 0.3	N/A	N/A	N/A
***a*L199**	N/A	N/A	N/A	N/A	N/A	N/A	5.6 ± 0.7	5.9 ± 0.8	5.7 ± 0.7
***a*F213**	N/A	N/A	N/A	N/A	N/A	N/A	3.8 ± 0.3	3.7 ± 0.3	3.8 ± 0.4
***a*P214**	N/A	N/A	N/A	N/A	N/A	N/A	4.6 ± 0.5	4.8 ± 0.6	5.0 ± 0.9
***a*V217**	N/A	N/A	N/A	N/A	N/A	N/A	3.7 ± 0.3	3.6 ± 0.3	3.6 ± 0.2
***a*W218**	N/A	N/A	N/A	N/A	N/A	N/A	2.3 ± 0.4	2.4 ± 0.6	2.3 ± 0.4
***a*F221**	N/A	N/A	N/A	N/A	N/A	N/A	2.9 ± 0.4	2.9 ± 0.4	2.9 ± 0.4

c) TBAJ-876 (deprotonated)	*c*-ring			Lagging			Leading		
	**10-40 ns**	**40-70 ns**	**70-100 ns**	**10-40 ns**	**40-70 ns**	**70-100 ns**	**10-40 ns**	**40-70 ns**	**70-100 ns**
***c*A28**	5.0 ± 0.5	5.0 ± 0.5	5.0 ± 0.5	5.0 ± 0.5	5.0 ± 05	5.0 ± 0.5	5.2 ± 0.6	5.0 ± 0.7	5.5 ± 0.6
***c*V61**	5.0 ± 0.5	4.9 ± 0.6	4.8 ± 0.6	4.5 ± 0.5	4.6 ± 0.5	4.7 ± 0.5	5.0 ± 0.8	5.2 ± 1.0	5.1 ± 0.9
***c*G62**	4.1 ± 0.3	4.1 ± 0.3	4.1 ± 0.4	4.0 ± 0.2	4.1 ± 0.2	4.1 ± 0.3	4.4 ± 0.3	4.6 ± 0.4	4.5 ± 0.4
***c*L63**	4.0 ± 0.3	4.0 ± 0.3	4.0 ± 0.3	4.0 ± 0.3	4.0 ± 0.3	4.0 ± 0.3	4.3 ± 0.8	4.5 ± 0.9	4.5 ± 0.9
***c*E65**	3.5 ± 0.1	3.5 ± 0.1	3.5 ± 0.1	3.5 ± 0.1	3.5 ± 0.1	3.5 ± 0.1	3.5 ± 0.2	3.5 ± 0.2	3.5 ± 0.2
***c*A66**	3.6 ± 0.2	3.6 ± 0.2	3.6 ± 0.2	3.6 ± 0.2	3.6 ± 0.2	3.6 ± 0.2	3.6 ± 0.2	3.6 ± 0.2	3.6 ± 0.2
***c*A67**	3.8 ± 0.2	3.9 ± 0.3	3.9 ± 0.3	3.6 ± 0.2	3.6 ± 0.2	3.7 ± 0.2	3.9 ± 0.3	4.0 ± 0.3	3.9 ± 0.3
***c*Y68**	3.8 ± 0.2	3.8 ± 0.2	3.8 ± 0.2	3.8 ± 0.2	3.8 ± 0.2	3.8 ± 0.2	3.8 ± 0.2	3.8 ± 0.3	3.8 ± 0.3
***c*F69**	3.4 ± 0.2	3.4 ± 0.2	3.4 ± 0.2	3.4 ± 0.2	3.4 ± 0.2	3.4 ± 0.2	3.5 ± 0.2	3.4 ± 0.2	3.4 ± 0.2
***c*I70**	3.6 ± 0.2	3.6 ± 0.2	3.6 ± 0.2	3.6 ± 0.2	3.6 ± 0.2	3.7 ± 0.3	3.6 ± 0.2	3.6 ± 0.3	3.7 ± 0.3
***c*L72**	3.8 ± 0.7	3.8 ± 0.7	3.9 ± 0.8	3.5 ± 0.3	3.5 ± 0.3	3.4 ± 0.3	4.4 ± 0.9	4.5 ± 0.9	4.7 ± 0.9
***c*F74**	5.3 ± 1.1	5.6 ± 1.2	6.0 ± 1.4	4.8 ± 0.7	5.2 ± 1.2	5.4 ± 1.2	6.7 ± 1.8	7.0 ± 2.2	7.0 ± 2.2
***c*E65:Oεx-LIG:N**	2.7 ± 0.1	2.7 ± 0.1	2.7 ± 0.1	2.7 ± 0.1	2.7 ± 0.1	2.7 ± 0.1	2.7 ± 0.1	2.7 ± 0.1	2.7 ± 0.1
***a*F169**	N/A	N/A	N/A	4.4 ± 0.8	4.9 ± 1.0	4.3 ± 0.8	N/A	N/A	N/A
***a*L170**	N/A	N/A	N/A	4.0 ± 0.4	4.0 ± 0.4	4.1 ± 0.5	N/A	N/A	N/A
***a*P172**	N/A	N/A	N/A	3.5 ± 0.3	3.6 ± 0.3	3.6 ± 0.3	N/A	N/A	N/A
***a*I173**	N/A	N/A	N/A	3.6 ± 0.2	3.5 ± 0.2	3.6 ± 0.2	N/A	N/A	N/A
***a*V176**	N/A	N/A	N/A	3.7 ± 0.3	3.8 ± 0.3	3.8 ± 0.3	N/A	N/A	N/A
***a*L199**	N/A	N/A	N/A	N/A	N/A	N/A	5.2 ± 0.9	5.0 ± 1.3	4.4 ± 1.4
***a*F213**	N/A	N/A	N/A	N/A	N/A	N/A	3.6 ± 0.3	3.7 ± 0.4	4.0 ± 0.7
***a*P214**	N/A	N/A	N/A	N/A	N/A	N/A	4.2 ± 0.5	4.5 ± 0.7	4.3 ± 0.5
***a*V217**	N/A	N/A	N/A	N/A	N/A	N/A	3.8 ± 0.3	3.8 ± 0.3	3.9 ± 0.5
***a*W218**	N/A	N/A	N/A	N/A	N/A	N/A	2.3 ± 0.4	2.4 ± 0.5	2.5 ± 0.6
***a*F221**	N/A	N/A	N/A	N/A	N/A	N/A	3.1 ± 0.4	3.1 ± 0.5	3.2 ± 0.5

**d) TBAJ-876 (protonated)**	***c*-ring**			**Lagging**			**Leading**		
	**10-40 ns**	**40-70 ns**	**70-100 ns**	**10-40 ns**	**40-70 ns**	**70-100 ns**	**10-40 ns**	**40-70 ns**	**70-100 ns**
***c*A28**	5.1 ± 0.5	5.1 ± 0.6	5.2 ± 0.7	4.9 ± 0.6	4.8 ± 0.5	4.8 ± 0.6	5.2 ± 0.5	5.2 ± 0.7	5.6 ± 0.8
***c*V61**	4.9 ± 0.6	4.8 ± 0.6	4.7 ± 0.6	5.0 ± 0.5	5.4 ± 0.7	5.5 ± 0.7	5.7 ± 0.5	5.3 ± 0.8	5.5 ± 1.0
***c*G62**	4.2 ± 0.3	4.2 ± 0.4	4.1 ± 0.5	4.2 ± 0.3	4.4 ± 0.4	4.5 ± 0.5	4.4 ± 0.3	4.4 ± 0.4	4.6 ± 0.6
***c*L63**	4.0 ± 0.4	4.0 ± 0.4	3.9 ± 0.3	4.2 ± 0.3	4.3 ± 0.4	4.8 ± 0.7	4.4 ± 0.5	4.2 ± 0.4	4.4 ± 0.8
***c*E65**	3.5 ± 0.1	3.5 ± 0.1	3.5 ± 0.1	3.5 ± 0.1	3.5 ± 0.1	3.6 ± 0.2	3.6 ± 0.2	3.5 ± 0.1	3.6 ± 0.2
***c*A66**	3.6 ± 0.2	3.6 ± 0.2	3.6 ± 0.3	3.6 ± 0.2	3.6 ± 0.2	3.6 ± 0.2	3.6 ± 0.2	3.6 ± 0.2	3.6 ± 0.2
***c*A67**	3.8 ± 0.2	3.8 ± 0.2	3.8 ± 0.2	3.7 ± 0.2	3.7 ± 0.2	3.8 ± 0.2	3.9 ± 0.3	3.9 ± 0.3	4.2 ± 0.4
***c*Y68**	3.8 ± 0.2	3.8 ± 0.2	3.8 ± 0.2	3.7 ± 0.2	3.7 ± 0.2	3.7 ± 0.2	3.7 ± 0.2	3.8 ± 0.2	3.7 ± 0.2
***c*F69**	3.6 ± 0.2	3.6 ± 0.2	3.6 ± 0.2	3.6 ± 0.2	3.6 ± 0.2	3.6 ± 0.2	3.4 ± 0.2	3.6 ± 0.2	3.6 ± 0.2
***c*I70**	3.7 ± 0.3	3.7 ± 0.3	3.7 ± 0.3	3.6 ± 0.2	3.6 ± 0.2	3.6 ± 0.2	3.5 ± 0.2	3.6 ± 0.2	3.9 ± 0.5
***c*L72**	5.0 ± 1.2	5.1 ± 1.3	5.2 ± 1.4	3.9 ± 0.3	3.8 ± 0.3	3.9 ± 0.3	3.9 ± 0.6	4.5 ± 0.7	5.1 ± 1.5
***c*F74**	7.1 ± 1.2	7.1 ± 1.2	7.1 ± 1.3	5.5 ± 0.6	5.0 ± 0.7	5.0 ± 0.6	4.9 ± 1.1	6.9 ± 0.8	7.1 ± 1.5
***c*E65:Oεx-LIG:N**	2.7 ± 0.1	2.7 ± 0.1	2.7 ± 0.1	2.7 ± 0.1	2.7 ± 0.1	2.7 ± 0.1	2.8 ± 0.1	2.7 ± 0.1	2.8 ± 0.1
***a*F169**	N/A	N/A	N/A	3.6 ± 0.2	5.6 ± 1.4	5.1 ± 1.4	N/A	N/A	N/A
***a*L170**	N/A	N/A	N/A	4.3 ± 0.5	4.3 ± 0.5	4.5 ± 0.6	N/A	N/A	N/A
***a*P172**	N/A	N/A	N/A	3.9 ± 0.3	3.9 ± 0.3	3.9 ± 0.3	N/A	N/A	N/A
***a*I173**	N/A	N/A	N/A	3.7 ± 0.2	3.6 ± 0.2	3.7 ± 0.2	N/A	N/A	N/A
***a*V176**	N/A	N/A	N/A	3.8 ± 0.3	3.8 ± 0.3	3.7 ± 0.3	N/A	N/A	N/A
***a*L199**	N/A	N/A	N/A	N/A	N/A	N/A	5.1 ± 1.3	5.4 ± 0.9	5.8 ± 1.1
***a*F213**	N/A	N/A	N/A	N/A	N/A	N/A	3.9 ± 0.5	4.1 ± 0.5	4.1 ± 0.6
***a*P214**	N/A	N/A	N/A	N/A	N/A	N/A	4.1 ± 0.4	5.2 ± 0.7	5.9 ± 1.2
***a*V217**	N/A	N/A	N/A	N/A	N/A	N/A	4.3 ± 0.7	3.8 ± 0.3	3.8 ± 0.3
***a*W218**	N/A	N/A	N/A	N/A	N/A	N/A	2.9 ± 1.1	2.2 ± 0.5	2.4 ± 0.5
***a*F221**	N/A	N/A	N/A	N/A	N/A	N/A	2.9 ± 0.5	3.3 ± 0.4	3.5 ± 0.5

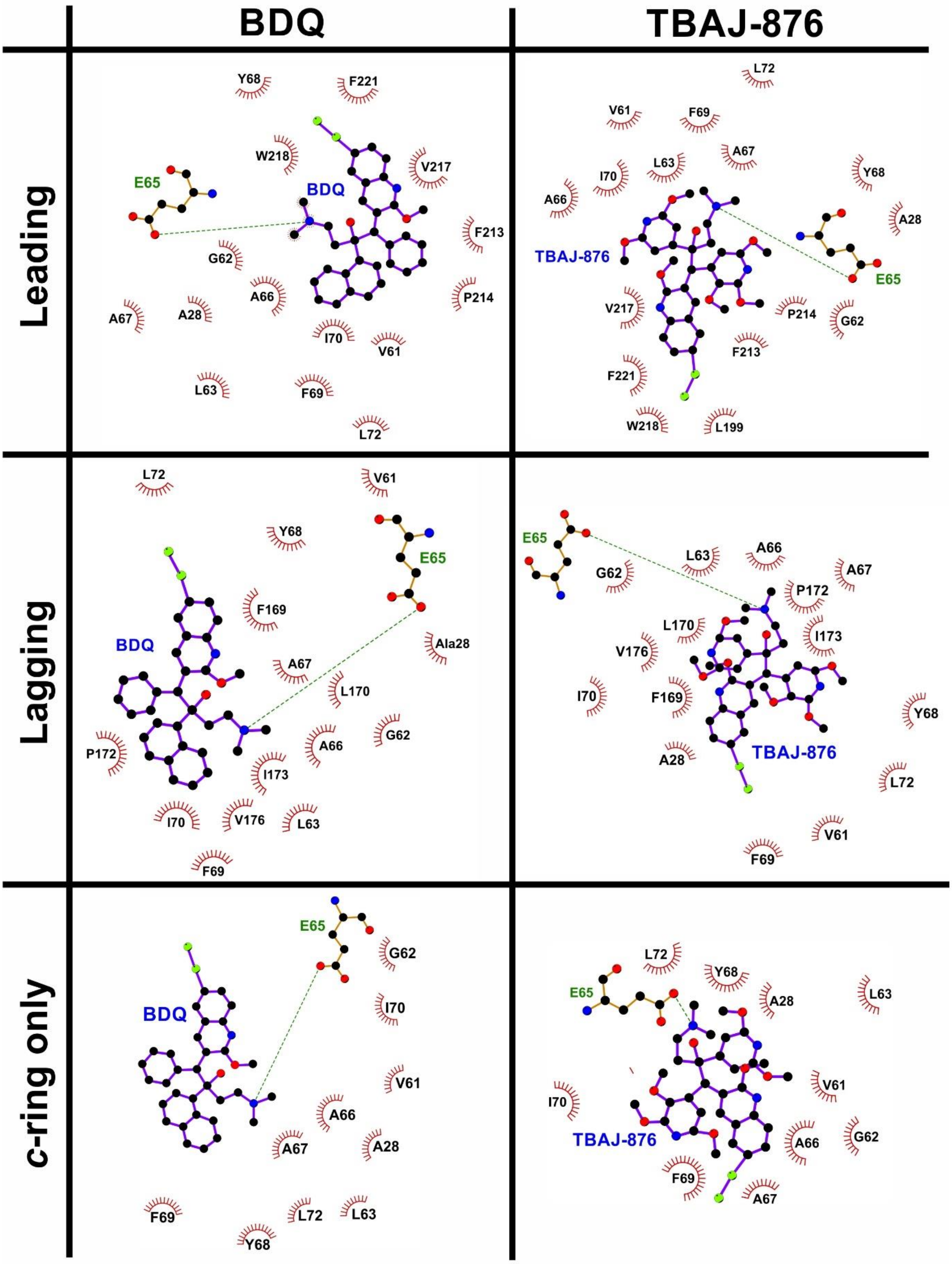

Figure S1: Protein drug interactions. LigPlot+ representations of protein-drug interactions for both drugs bound, when E65 and the drugs are charged, to all three sites. Carbon, nitrogen, oxygen and bromine (also lone pair) atoms of the drug are shown in *black*, *blue*, *red* and *green*, respectively.
